# Increase of 10% in the Rate of Adverse Drug Reactions for Each Drug Administered in Hospitalized Patients

**DOI:** 10.6061/clinics/2018/e185

**Published:** 2018-01-17

**Authors:** Marisa Rosimeire Ribeiro, Antonio Abílio Motta, Luiz Augusto Marcondes-Fonseca, Jorge Kalil-Filho, Pedro Giavina-Bianchi

**Affiliations:** I Servico de Imunologia Clinica e Alergia, Hospital das Clinicas HCFMUSP, Faculdade de Medicina, Universidade de Sao Paulo, Sao Paulo, SP, BR; II Instituto Nacional de Ciencia e Tecnologia (INCT) de Investigacao em Imunologia, Sao Paulo, SP, BR

**Keywords:** Adverse Drug Reactions, Hypersensitivity Reactions, In-patients, Incidence, Risk factors

## Abstract

**OBJECTIVE:**

To assess the risk factors, incidence and severity of adverse drug reactions in in-patients.

**METHODS:**

This prospective study evaluated 472 patients treated at a teaching hospital in Brazil between 2010 and 2013 by five medical specialties: Internal Medicine, General Surgery, Geriatrics, Neurology, and Clinical Immunology and Allergy. The following variables were assessed: patient age, gender, comorbidities, family history of hypersensitivity, personal and family history of atopy, number of prescribed drugs before and during hospitalization, hospital diagnoses, days of hospitalization. The patients were visited every other day, and medical records were reviewed by the investigators to detect adverse drug reactions.

**RESULTS:**

There were a total of 94 adverse drug reactions in 75 patients. Most reactions were predictable and of moderate severity. The incidence of adverse drug reactions was 16.2%, and the incidence varied, according to the medical specialty; it was higher in Internal Medicine (30%). Antibiotics were the most commonly involved medication. Chronic renal failure, longer hospital stay, greater number of diagnoses and greater number of medications upon admission were risk factors. For each medication introduced during hospitalization, there was a 10% increase in the rate of adverse drug reaction. In the present study, the probability of observing an adverse drug reaction was 1 in 104 patients per day.

**CONCLUSIONS:**

Adverse drug reactions are frequent and potentially serious and should be better monitored in patients with chronic renal failure or prolonged hospitalization and especially in those on ‘polypharmacy’ regimens. The rational use of medications plays an important role in preventing adverse drug reactions.

## INTRODUCTION

Adverse drug reactions (ADRs) in hospitalized patients can be divided into two categories: those that are the cause of hospital admission, and those that occur during hospitalization 1. There are limited data on ADRs, especially regarding the reactions that occur after admission. It is estimated that ADRs occur in 10% of the general population and 10 to 20% of in-patients 1,2. Approximately 15 to 20% of ADRs correspond to hypersensitivity drug reactions (HDRs), which are induced by exposure to a drug in a dose that is usually tolerated by healthy individuals, and the reactions are characterized by objective symptoms that can be reproduced following subsequent re-exposure 2, 3.

A meta-analysis of 33 prospective studies from the United States, between 1966 and 1996, showed that 15.1% of hospitalized patients suffered an ADR and 0.32% of patients hospitalized died because of an ADR, with an estimated 106,000 deaths during this period 4. There are questions about the methodology and validity of this study because of its heterogeneity, including differences in population, surveillance techniques and ADR definitions 5. In England, a study evaluating 18,820 patients, has found that 6.5% of hospitalizations were directly caused by an ADR, and another research showed that 15.8% of hospitalized patients developed an ADR 1, 6.

With the current demographic changes, such as an aging population, in addition to changes in clinical practice that have occurred in recent decades, there is a need for further studies on ADRs 1. The detection of ADRs in hospitals is an important measure of morbidity associated with drug use, and their burden on health system 7. The incidence of hospital ADRs is a key parameter for determining the quality of care. Furthermore, inadequate information about ADR rates is the most important factor in the failure to adopt measures that have a significant impact on patient safety 8.

In-patients represent a population of special interest because ADRs can be observed directly in these patients, who usually have many comorbidities and require treatment with special medications 9. The present study assessed the clinical and demographic features of patients with ADRs, identifying the risk factors associated with the reactions.

## METHOD

### Study design and subjects

This observational and prospective study was performed from February 2010 to December 2013 in five medical specialty infirmaries specialties at the Hospital das Clínicas da Faculdade de Medicina da Universidade de São Paulo (HCFMUSP), a tertiary teaching hospital. The medical specialties involved in the study were: Internal Medicine, Surgery, Neurology, Geriatrics, and Immunology. The study protocol was approved by the HCFMUSP Ethics Committee (Protocol: 550/08) and was in accordance with the Helsinki Declaration of 1975. All patients provided written informed consent.

A convenience sample was selected to form a prospective cohort. Sampling occurred on random days and ended when we recruited the target number of patients (n=100) from each infirmary.

The follow-up was performed from study inclusion until hospital discharge. The included were at least 18 years of age, represented both genders, had a minimum hospital stay of 48 hours, and had the cognitive ability to answer the study questionnaires. Patients with an ADR as the cause of hospitalization were excluded. If there were repeated hospitalizations for a single patient, only data from the first admission were included.

### Procedures

Participants were interviewed with the questionnaire suggested by ENDA (*European Network for Drug Allergy*) 10,11. A questionnaire assessing asthma and atopy, developed from GINA (*Global Initiative for Asthma*) and ISAAC (*International Study of Asthma and Allergies in Childhood*), was also used 12-14.

All questionnaires were administered by a single researcher, MRR, who is a specialist in clinical immunology and allergy, with expertise in drug adverse reactions. After the first interview, visits on alternate days were conducted by the same researcher to monitor patient records, laboratory exams, and prescriptions. Patients in each infirmary were followed until discharge. All patients were instructed to contact or visit their medical specialty if they had any reaction within 30 days after hospital discharge. The medical staff at the medical specialties were oriented about the study and could directly inform the researchers when there was an ADR.

Admission to the hospital was considered the exposure and the presence or absence of ADRs was the outcome. The definition suggested by the World Health Organization for an ADR is: “undesirable and unintended reactions that occur due to the use of a particular medication at pharmacological doses for therapy, prophylaxis or diagnosis” 15. HDRs, a subgroup of ADRs, which are based on the individual’s predisposition, include unexpected reactions initiated by exposure to a drug, in a dose usually tolerated by healthy individuals, and characterized by objective signs and symptoms that could be reproduced following re-exposure 3. The scale of probability (Naranjo et al.) was used to establish a causal relationship and classify the ADRs as ‘possible, probable or definite’ 16. Any ADRs that were classified as ‘doubtful’ were excluded. We classified the ADRs as A (predictable) and B (unpredictable), according to criteria developed by Rawlins and Thompson 17. The ADRs were scored according to severity using the criteria of Hartwig et al. (i.e., “mild, moderate or severe”) 18. All classifications were made by consensus of three researchers, MRR, AAM, and PGB.

The following qualitative variables were assessed: gender, race, age, family history of HDRs, personal and/or family history of atopy, asthma diagnosis, chronic renal failure, congestive heart failure, liver disease, human immunodeficiency virus (HIV) infection, and chronic urticaria. The quantitative variables that were measured included the number of comorbidities, number of hospital diagnoses, number of medications before and during hospitalization, and number of days of hospitalization. The medical specialties were compared using qualitative and quantitative variables.

### Statistical Analysis

Patient characteristics were described using absolute and relative frequencies for the qualitative variables, and summary measures (mean, standard deviation, median, P25 and P75) for the quantitative variables. The associations between qualitative variables and the medical specialties were checked using chi-square tests, or tests of likelihood ratio, according to Kirkwood and Sterne 19. Quantitative characteristics were compared using the Kruskal-Wallis test, followed by the Dunn multiple comparison (except for ages, which were compared using ANOVA, followed by Tukey’s multiple comparisons) 20.

The chi-square test of likelihood ratio test or the Fisher exact test was used to identify possible associations between qualitative variables. Quantitative variables, according to the occurrence of reactions, were compared using the Mann-Whitney U-test, except for age, which was compared using Student’s t-test 19. The odds ratios of each variable of interest associated with the general ADRs and HDRs, with confidence intervals of 95%, were calculated using simple logistic regression, which was performed to quantify the isolated influence of each feature in the adverse reaction events 21.

Multiple logistic regression models were estimated for the general ADRs by selecting the variables that showed levels of significance lower than 0.20 (*p*<0.20) in the bivariate tests, and lower than 0.05 (*p*<0.05) for the HDRs because of the low number of hypersensitivity reactions 21.

## RESULTS

A total of 472 patients were selected and 8 (1.7%) were hospitalized due to ADRs. Therefore, the prevalence of ADRs as a cause of hospitalization was 1.7%. We included 464 patients distributed as follows: 100 patients each in Geriatrics, Internal Medicine and General Surgery, and 103 patients in Neurology. Immunology included only 61 patients, due to a large number of readmissions.

The average age of the patients included in the study was 57.5 years (SD±19.9), and 239 (51.5%) were female. The mean hospital stay was 16.8 days, and the average number of medications used during hospitalization was 15.5 medications per patient.

**Figure 1:**
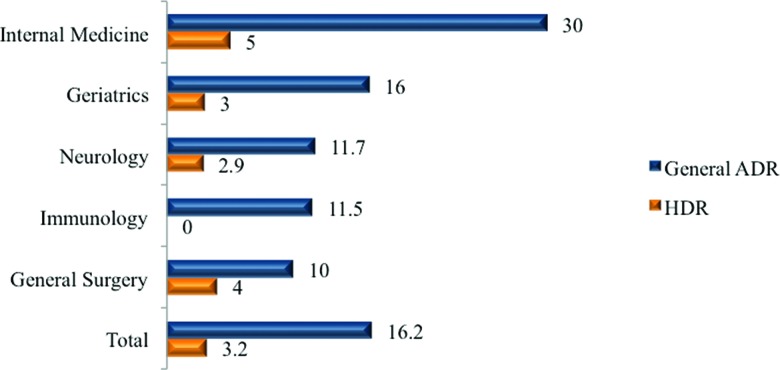
Incidence of ADRs and HDRs by medical specialty (%). ADR: Adverse drug reaction HDR: Hypersensitivity drug reaction. The ADR rate was higher in Internal Medicine (*p*=0.001).

We observed 94 ADRs in 75 patients who had at least one adverse reaction. The overall incidence of ADRs during hospitalization was 16.2%, and the incidence of ADRs according to the medical specialty varied; the incidence was higher in Internal Medicine (Figure 1) (*p*=0.001). There was no difference in the incidence of HDRs between medical specialties (*p*=0.278).

There were more ADRs in patients with chronic renal failure (*p*=0.012), prolonged hospital stays (*p*<0.001), higher number of diagnoses (*p*<0.001), and higher number of medications used during their hospital stay (*p*<0.001). After multiple logistic regression models were applied, it was found that for each medication introduced during hospitalization, there was a 10% increase in the rate of an overall adverse event (*p*<0.001), independent of other patient characteristics. Similarly, the increase of one medication during hospitalization caused a 14% increase in the chance of HDR (*p*<0.001). There was a 57% less chance of new ADRs in patients with a previous history of ADRs (*p*=0.017) (Table 1).

The predictable ADRs, type A, were most frequently observed (84% of ADRs), with gastrointestinal manifestations being the most common (Table 2). According to the criteria developed by Naranjo et al., most of the ADRs were classified as “possible or likely” because skin and/or provocation tests were not performed to confirm a causal relationship 16.

**Figure 2:**
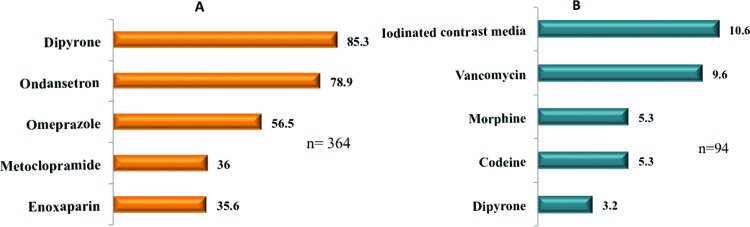
Most frequently prescribed medications (A) and medications associated with ADRs (B). ADR: Adverse drug reactions.

Symptomatic medications were the most commonly prescribed. The primary medication groups that caused ADRs were antibiotics (21.2%), followed by opioids (13.8%), and iodinated contrast media (10.6%) (Figure 2). Seven patients developed ADR due to drug interactions (Table 2). Skin eruptions were the most common HDR (53.3%), followed by hematologic (26.6%) and gastrointestinal manifestations (13.3%); one patient had a systemic reaction (anaphylaxis). There was no significant difference in the frequency of immediate (i.e., occurring within 1 hour after the drug administration) or non-immediate HDRs.

ADR incidence density varied between the medical specialties. However, when considered collectively, we observed one patient with an ADR per 104 patients per day. The probability of observing one HDR was one for every 500 patients in any one day (Table 3). According to the severity level described by Hartwig et al. 18, most ADRs were moderate (44.7%), requiring treatment and/or extension of hospital stay, followed by mild (42.5%), and severe (12.8%).

## DISCUSSION

The prevalence of ADRs in this study as a cause of hospitalization was 1.7%, which is similar to another Brazilian study, which observed an ADR prevalence of 2.1% in 10,272 patients 22. Higher rates, 3.3% in 4,332 patients and 6.5% in 18,820, have been shown in two European surveys. While the objective of these studies were to assess the prevalence of ADRs as the cause of hospital admission 6,23, we analyzed hospitalized patients in specific clinical areas.

We observed an overall ADR incidence of 16.2%, with variations in the different medical specialties that were analyzed, and the incidence was higher in Internal Medicine (30%). The overall incidence density observed showed the likelihood of an ADR was one in every 104 patients hospitalized in any one day. Another study evaluated 3,695 patients and also found differences across 12 medical specialties 1.

Several studies have shown similar values to ours, in terms of general ADR incidence (between 10% and 15.8%) 1,4,23-25, except for one study that observed a rate of 31% 7. In terms of the Internal Medicine results, our incidence was similar to the rate observed in another study, which found an incidence of 22% 26. The discrepancy observed between studies may be explained by differences in the study populations and the ADR definitions used.

There are limitations related to the Naranjo criteria, which are intended to assess the likelihood of an ADR associated with only one drug, and confounding variables can compromise the sensibility and specificity of this method, such as other algorithms. However, these criteria are used by many authors when reporting drug interactions and are recommended by several journals to reviewers of manuscripts. In a study comparing three different pharmacovigilance algorithms (Kramer algorithm, Naranjo criteria and Jones algorithm) used to assess the likelihood of an ADR in the intensive care unit showed similar results, suggesting that the selection of any of these three instruments is reasonable 27.

Chronic renal failure patients had more ADRs, as did patients with longer hospitalization stays, a greater number of diagnoses, and greater number of medications used at admission. The number of medications used was the only independent risk factor for ADRs observed in this study, which is similar to the results from another study 1. The largest number of diagnoses during hospitalization was observed in the Internal Medicine and Geriatric infirmaries, reflecting the highest number of drugs prescribed in these medical specialties, and the highest incidence of ADRs was in Internal Medicine patients. Age was not an isolated risk factor for ADRs, differing from other studies 7,23,26. A history of previous ADR was a protective factor for the occurrence of new ADRs (p=0.017), probably because the awareness ‘prevents’ further exposure to certain medication classes. The only independent risk factor for HDR during hospitalization was the number of medications on admission (p<0.001), and each new medication prescribed for patients during hospitalization, increased the rate of HDR by 14%.

Type A reactions were the most common (84% of ADR), particularly gastrointestinal manifestations, as in other studies 7,23-25,28-29. The incidence of skin reactions was 2%, which is higher than the rate of 0.7% reported in another study 30. In HDRs, skin manifestations were the most common presentations restricted to one organ (53.3%). Most observed ADRs were classified as moderate, with high morbidity, in accordance with other studies 26,29. The rate of 12.8% for severe reactions observed in the present study is also consistent with the literature 1,25,31.

Symptomatic medications were the most prescribed agents during hospitalization, highlighted in descending order: dipyrone, omeprazole and metoclopramide. Dipyrone use is not permitted in many countries where paracetamol is identified as the most prescribed alternative 23. Antibiotics caused the most ADRs and HDRs (21.2%), followed by opioids and iodinated contrast agents, which aligns with current literature data 2,28.

ADRs are common and potentially serious events that should be monitored through pharmacovigilance interventions that increase the number of notifications because many events are not reported spontaneously. Our study contributes further evidence to warn health professionals about the important role of rational use of medications and to support the prevention of ADRs, a significant problem into health systems, particularly in patients with chronic renal failure, prolonged hospitalization, or those on “polypharmacy” medicine regimens.

## Figures and Tables

**Table 1:** Odds ratio of experiencing an ADR by medical specialty, history of previous ADR and number of medications.

Variable	OR (95% CI)	*p*
**Medical Specialty**	**OR (95% CI) in the Internal Medicine infirmary**	
Internal Medicine	1.00	
Geriatrics	0.37 (0.18-0.78)	**0.009**
Neurology	0.25 (0.11-0.56)	**0.001**
General Surgery	0.17 (0.07-0.4)	**<0.001**
Immunology	0.50 (0.019-1.34)	0.170
**Previous ADR**	**OR (95% CI) in relation to No Previous ADR**	
No	1.00	
Yes	0.43 (0.23-0.8)	**0.008**
**Number of medications**		
One mediation added	1.1 (1.06-1.13)	**<0.001**

ADR: Adverse drug reaction.

**Table 2:** ADRs and related drugs.

ADR (n=94)	Related drugs/number of reactions
**Cardiovascular (4)**	
Acute atrial fibrillation	Formoterol+Fenoterol (1)
Arterial hypertension	Prednisone (1)
Postural hypotension	Fentanyl (1) Hydrochlorothiazide (1)
**Gastrointestinal (26)**	
Constipation	Codeine (3) Tramadol (1)
Nausea and vomiting	Potassium chloride (1) Mannitol (1) Iodinated contrast media (3) Dipyrone (1) Tramadol (1) calcium gluconate (1) Morphine (1) Prednisone (1) Clindamycin (1) Calcium polystyrene sulfonate (1)
Epigastric pain	Lactulose (1) Iron sulfate (1)
Diarrhea	Bromopride (1) Iodinated contrast media (2) Omeprazole (1)
Abdominal cramps	Vitamin B12 (1)
Hepatitis	Ampicillin (1)*
Cholestasis	Fluconazole (1)
Pancreatitis	Furosemide (1)*
**Infectious (3)**	
Pseudomembranous colitis	Imipenem (1) Ciprofloxacin (1) Piperacillin/tazobactam (1)
**Neurological (12)**	
Tremors	Oxacillin (1)
Malaise, dizziness	Metoclopramide (1)
Headache, dizziness and vomiting	Carbamazepine (1)
Somnolence	Haloperidol (1) Bromazepam (1)
Lowering NC, miosis	Morphine (1)
Hallucinations and mental Confusion	Escitalopram (1) Piperacillin/tazobactam (1) Codeine (2) Meropenem (1)
Convulsion	Imipenem (1)
**Kidney (17)**	
Renal failure	Vancomycin (8) Captopril (1) Iodinated contrast media (1) Losartan (1) Amphotericin B (1) Furosemide (1) Meloxicam (1)Mannitol+Bisacodyl+Lactulose (2) Ipratropium +Tiotropium (1)
**Skin (10)**	
Erythroderma	Vancomycin (1)*
Rash and itchy skin	Cefazolin (1)*, Iodinated contrast media (1)*, Morphine (3)*
Maculopapular rash	Ketoprofen (1)*
Itching	Dipyrone (1) Gabapentin (1)
Angioedema + hives	Ceftriaxone (1)*
**Systemic (1)**	
Anaphylaxis	Iodinated contrast media (1)*
**Hematologic (9)**	
Hemolytic anemia	Ceftriaxone (1)*
Pancytopenia	Amphotericin B (1)*
Neutropenia	Dipyrone (1)*
Leukopenia	Cyclophosphamide+Vincristine (1)
Thrombocytopenia	Unfractionated Heparin (1)*
Bleeding	Warfarin (1) Clopidogrel+Acetilsalicilic acid+Enoxaparin (1) Acetilsalicilic acid+Enoxaparin (1) Unfractionated Heparin (1)
**Endocrine - Metabolic (10)**	
Hypotension and Hyponatremia	Furosemide (1)
Hyperkalemia	Enalapril (1) Losartan (1)
Hypokalemia	Furosemide (1)
Edema	Prednisone (1)
Hypoglycemia	Glargine insulin (1)
Hyperglycemia	Methylprednisolone (3) Prednisone (1)
**Other (2)**	
Thoracic pain, nausea	Iodinated contrast media (1)

* Hypersensitivity drug reaction.

**Table 3:** Incidence density of ADR and HDR by medical specialty.

Infirmary	ADR	Incidence density of ADR (cases/day)	Relative risk to ADR	HDR	Incidence density of HDR (cases/day)	Number of days of hospitalization
Internal Medicine	30	0.014	1.00	5	0.002	2107
Geriatrics	16	0.008	0.57	3	0.002	1975
Neurology	12	0.006	0.43	3	0.002	1934
General Surgery	10	0.008	0.57	4	0.003	1292
Immunology	7	0.014	1.00	0	0.000	504
**Total**	75	0.010	0.71	15	0.002	7812

ADR: Adverse drug reaction.

HDR: hypersensitivity drug reaction.
